# On-chip multiplexed single-cell patterning and controllable intracellular delivery

**DOI:** 10.1038/s41378-019-0112-z

**Published:** 2020-02-24

**Authors:** Zaizai Dong, Yanli Jiao, Bingteng Xie, Yongcun Hao, Pan Wang, Yuanyuan Liu, Junfeng Shi, Chandani Chitrakar, Stephen Black, Yu-Chieh Wang, L. James Lee, Mo Li, Yubo Fan, Lingqian Chang

**Affiliations:** 1 School of Biological Science and Medical Engineering, Beihang University, 100083 Beijing, China; 2 Institute of Nanotechnology for Single Cell Analysis (INSCA), Beijing Advanced Innovation Center for Biomedical Engineering, Beihang University, 100083 Beijing, China; 3 College of Agricultural and Life Sciences, University of Florida, Gainesville, FL 32611 USA; 4 Center for Reproductive Medicine, Peking University Third Hospital, 100191 Beijing, China; 5 Ministry of Education Key Laboratory of Micro and Nano Systems for Aerospace, Northwestern Polytechnical University, 710072 Xi’an, China; 6 Chemical and Biomolecular Engineering Department, Ohio State University, Columbus, OH 43209 USA; 7 Department of Biomedical Engineering, University of North Texas, Denton, TX 76207 USA; 8 Department of Pharmaceutical Sciences, University of North Texas Health Science Center, Fort Worth, TX 76107 USA

**Keywords:** Bionanoelectronics, Nanofabrication and nanopatterning, Nanostructures

## Abstract

Conventional electroporation approaches show limitations in the delivery of macromolecules in vitro and in vivo. These limitations include low efficiency, noticeable cell damage and nonuniform delivery of cells. Here, we present a simple 3D electroporation platform that enables massively parallel single-cell manipulation and the intracellular delivery of macromolecules and small molecules. A pyramid pit micropore array chip was fabricated based on a silicon wet-etching method. A controllable vacuum system was adopted to trap a single cell on each micropore. Using this chip, safe single-cell electroporation was performed at low voltage. Cargoes of various sizes ranging from oligonucleotides (molecular beacons, 22 bp) to plasmid DNA (CRISPR-Cas9 expression vectors, >9 kb) were delivered into targeted cells with a significantly higher transfection efficiency than that of multiple benchmark methods (e.g., commercial electroporation devices and Lipofectamine). The delivered dose of the chemotherapeutic drug could be controlled by adjusting the applied voltage. By using CRISPR-Cas9 transfection with this system, the *p62* gene and *CXCR7* gene were knocked out in tumor cells, which effectively inhibited their cellular activity. Overall, this vacuum-assisted micropore array platform provides a simple, efficient, high-throughput intracellular delivery method that may facilitate on-chip cell manipulation, intracellular investigation and cancer therapy.

## 3D Electroporation: Uniform and safe electricity-based drug delivery

Applying an electric field to individually trapped cells allows for the efficient and safe delivery of drugs or genes, and the characterization and manipulation of cellular activity. Electroporation is a technique that increases the permeability of a cell’s membrane, to allow the infiltration of drugs or genes. In practice, traditional electroporation has issues with efficiency and cellular damage. The scientists of Lingqian Chang (Beihang University) and Mo Li (Peking University Third Hospital) led a team to develop an on-chip “3D” system that uses a vacuum to trap cells in individual micropores. By applying an electric field, the team were able to deliver cargo of different sizes, from drugs to genes, into the cells with greater efficiency and safety than benchmark methods. The dose of chemotherapy drugs was controlled through altering the voltage applied. The team were able to inhibit tumor activity by delivering CRISPR-Cas9 gene editing plasmid with their system. This “3D” system was showed the potential for on-chip manipulation, in situ intracellular interrogation and cancer therapy.

## Graphical abstract

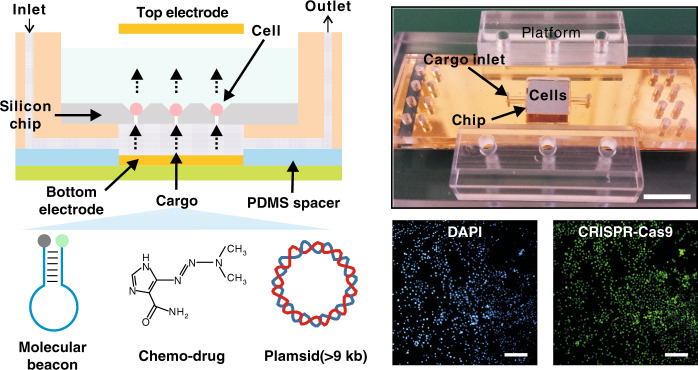

## Introduction

Electroporation, as a simple nonviral tool for gene delivery, has been commonly used for biomedical research^1^. Its potential use in vivo, including for regenerative medicine^2^, adoptive immunotherapy^3,4^, and gene editing^5^, has been explored recently. Compared to other physical methods (e.g., microinjection, sonoporation and optoporation), electroporation is easier to perform and can be controlled at the single-cell level because the lipid bilayer of the cell membrane is sensitive to the extracellular electric field^6,7^. In conventional electroporation designs, a couple of electrodes (which are many thousands of times larger than normal cells) are placed in a chamber where a high voltage (100 V or higher) current is applied to a large number of cells^8^. The expected disadvantages of using such a design include unrecoverable damage in cells and insufficient delivery efficiency. The outcomes of substance delivery, which could be highly variable among different cell types, cargo types and buffer solutions, may be hard to predict prior to experiments^9,10^.

To address these issues, 3D single-cell electroporation (3D EP) platforms have been developed^11–15^. The key component of these platforms is a film containing micropores or nanopores. The electric field applied across the film can be focused on cells that are aligned with the pores. By positioning cells within the microscale structure, the strength of the electric field localized to the cell membrane can be enhanced by several orders of magnitude compared to that of commercial electroporation devices^16^. At a voltage as low as 1 V, the permeabilization of the cell membrane to allow cargo delivery within such a confined area can be achieved while limiting the risk of damage to cells^6,17^. In addition, it is easy to adapt the electroporation system on the lab-chip for use in a variety of applications, ranging from molecular characterization (e.g., cell-cell interactions and cellular heterogeneity) to the manipulation of gene expression (e.g., transfection of plasmid DNA, siRNA and miRNA)^17–22^.

With 3D EP devices, a key factor in achieving high delivery efficiency is the cell-pore alignment, which determines whether the electric field can be precisely targeted to the cells^6^. Therefore, cell manipulation plays an important role in on-chip electroporation platforms. Recently, track-etched porous membranes have been used as an interface for electroporation-based cell transfection or sampling^13,14,23^. However, the random distribution of the pores over the membranes results in challenges in controlling the cell location, cell-pore alignment, and cell-pore ratio. Thus, changes in the method of cell seeding or manipulation cannot significantly improve the transfection efficiency on track-etched membranes. Researchers, including our group, have designed various on-chip manipulation techniques to support 3D EP, including magnetic tweezers (MTs), dielectrophoresis (DEP), and microfluidics^11,13,24–26^. However, instrument-dependent techniques (i.e., MTs and DEP) usually cause irreversible cell damage due to thermal issues, pH changes, and other changes in the microenvironment^27,28^. In addition, the fabrication of microfluidics on a 3D porous membrane as part of user-friendly biomedical devices remains challenging.

In this work, a pyramid pit micropore array was reported for the vacuum-assisted 3D EP platform. An easy-to-perform microfabrication protocol based on the wet etching method was established for the fabrication of a highly ordered 3D micropore array. To achieve high delivery efficiency, we designed a controllable vacuum setup to trap each single cell onto each micropore. We analyzed multiple factors, including the micropore diameter, negative pressure, and chip surface properties, that affected the cell trapping efficiency and vacuum-induced cell damage. We also tested different treatments of the chip surface to facilitate cell trapping.

By using our platform, cargoes with a range of molecular weights, from molecular beacons (MBs, 22-bp oligonucleotides) to plasmid DNA (>9 kb), were delivered successfully into targeted cells. The cell inhibition efficiency of small molecule chemo-drugs (e.g., dacarbazine) was significantly higher than that resulting from direct treatment with chemo-drugs and drugs encapsulated by Lipofectamine. The dose of chemo-drugs delivered into cells could also be controlled by tuning the voltage applied to the cells. Furthermore, the CRISPR-Cas9 system effectively knocked out the *p62* and *CXCR7* genes, which resulted in the effective inhibition of the growth of tumor cells. Compared to previously reported micropore devices, including commercial track-etched membranes^14,15,29^ and silicon chips^6,11,20^, highly ordered pyramid pit micropore arrays on a silicon chip were easily fabricated using a simple cleanroom protocol. In addition, the pyramid pit micropores allowed each single cell to be confined in an isolated microenvironment for electrotransfection at low voltage. Overall, our platform permits simple, efficient, and high-throughput delivery of macromolecules and small molecules into living cells without compromising their viability, which allows for on-chip manipulation, in situ intracellular interrogation and cancer therapy.

## Results and discussion

### Single-cell electroporation on a micropore array

**Fig. 1:**
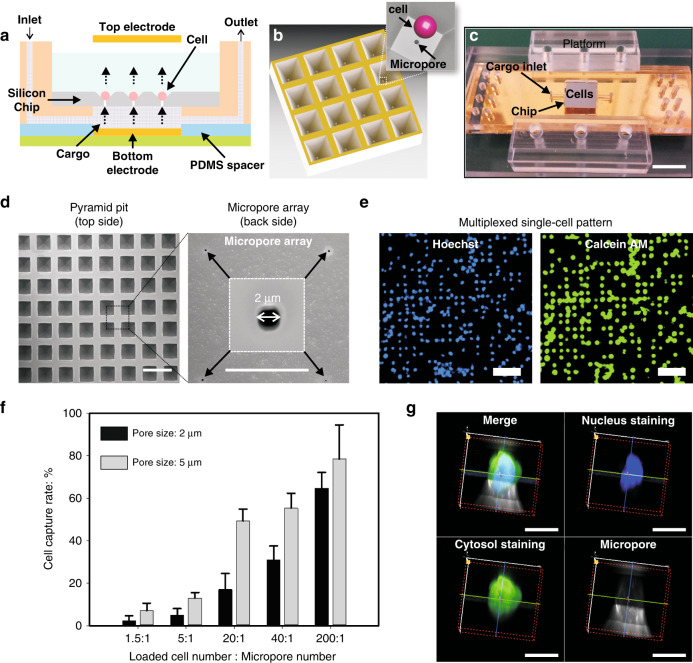
Use of the vacuum-assisted micropore array for multiplexed single-cell patterning. **a** A cross-sectional view of the 3D low-voltage electroporation (3D EP) platform setup. **b** A diagram of the pyramid pit-shaped micropore array chip. The zoomed-in view showed a single cell loaded on a micropore. **c** Photograph of the 3D EP platform. Scale bar: 1 cm. **d** Scanning electron microscopy showed both a pyramid pit array (top side) and a micropore array (back side). Scale bar: left 100 µm, right 20 µm. **e** Vacuum-assisted multiplexed single-cell array pattern on the chip. Scale bar: 100 µm. **f** The cell capture rate obtained with different ratios of the loaded cell number to the micropore number at a fixed pressure (1.5 psi). **g** 3D single-cell fluorescence model, as revealed by confocal microscopy, showing the percentage of the cell body inside the micropore (5 µm) after cell trapping. Scale bar: 10 µm.

We designed a new 3D EP platform based on a silicon chip, as shown in Fig. 1a–c. On the chip, each cell is gently trapped in a microwell by a vacuum. Genetic materials and small molecules are housed in a miniaturized chamber under the chip. The electric field for electroporation is established by a top plate electrode, which is made of gold-coated glass in cell buffer, and a bottom electrode made of the same material under the cargo-loading chamber. Based on the schematic of the electric field shown in Figs. S1–S4, compared with the flat micropore support, our pyramid pit micropore array guaranteed a better alignment, which allowed proper contact between the cells and the micropores and thus ensured a focused electrical field at the cell membrane and efficient electroporation with limited perturbation of cells^17^. Following electroporation, cargoes can be transported electrophoretically into cells through each micropore when the electric field remains activated (Fig. 1a). The 3D EP platform can achieve high-throughput electroporation, since more than 100,000 cells can be electroporated on one chip in parallel. Each cell was cultured in a microwell environment during posttransfection monitoring (Fig. 1b, c). In this work, mRNA regulation, chemo-drug responses and gene therapy were observed on-chip in real-time.

The simple implementation of a silicon chip-based device for cell patterning and electroporation was shown by our design. A pyramid pit microwell array was formed by the anisotropic etching silicon in a solution of 45% potassium hydroxide at 80 °C. The depth of the pyramid pit was controlled by the size of the etching mask (SiO_2_, 100 nm thick) due to the fixed angle (54.74°) of the mask used for etching (Fig. 1d, left panel). By controlling the etching period, open micropores were created on the bottoms of the pyramid pits. The micropore array on the back of chip was shown in the right panel of Fig. 1d. In our experiment, the thickness of the marginal silicon was 300 μm, while the thickness of the micropore array was 35 μm in the central region (see Materials and methods section). The microfabrication protocol was illustrated in Fig. S5.

### Vacuum-assisted cell patterning

By using the design shown in Fig. S6, we also improved the approach used for capturing cells on the chip. The nozzle of the filter chamber was made in house and was connected to a gauged vacuum pump. When negative pressure was generated inside the chamber, the buffer containing cells (liquid waste) flowed into the chamber through micropores and drove cells onto the micropore array by hydrodynamic force. The cells were aligned with the micropores under negative pressure, which guaranteed that the electric field could be more effectively applied to the cells and thus achieved low-voltage and high-efficiency electroporation (Figs. S1, S2). This was different from intracellular delivery based on the mechanical deformation of the cell membrane caused by cells passing through a long, narrow microchannel^30–33^. BEAS-2B cells (human bronchial epithelium) were used to test the vacuum-assisted cell trapping. At a given pressure, the ratio between the loaded cell number and the micropore number was critical to the final capture rate. By fixing this ratio at 200:1 (loaded cell number: micropore number), we obtained ~64.7% capture rate on the 2 μm micropores and 78.3% on the 5 μm micropores (Fig. 1e, f). The negative pressure was set to 1.5 psi, as a result, vacuum-induced cellular damage was not observed. However, such a high ratio (200:1) may not be feasible for rare cell populations or single-cell studies, as more than 99% of cells would be lost. Therefore, we continued to optimize the cell-trapping procedure (including the pore size, vacuum force, and chemical properties of the chip surface) with the ratio fixed at 1:1. The chemical properties of the chip surface have been reported as an important factor relevant to cell attachment and culture^34,35^. Therefore, we treated our chip with surface coatings of gelatin, bovine serum albumin (BSA) and polyethylene glycol (PEG). Under a vacuum pressure of 1.5 psi, the cell-trapping rate of the surface coated with BSA or gelatin was significantly higher than that of the control chip without any surface treatment (Fig. S7). The PEG-modified surface did not show a significantly improved cell-trapping rate. Notably, we achieved a maximum trapping efficiency of 75.6% and an average trapping efficiency of ~55% under vacuum pressures ranging from 7.5 to 12 psi. The pressure on each cell due to the vacuum was low (~3.25 × 10^−5^ N at 7.5 psi) and did not cause noticeable damage to the cells.

We examined the cell damage potentially caused by vacuuming micropores using fluorescence confocal microscopy. As shown in Fig. 1g, we measured the percentage of the cell body inside the micropore by labeling the cells with fluorescence dyes. Our results showed that the micropore size significantly affected cell viability (Fig. S8a). For BEAS-2B cells with an average size of 25 μm, micropores with 7 μm opening could aspirate more than 30% of the cell body inside the pore, which inevitably compromised the cell viability (~75%) (Fig. S8b). Based on our findings, we suggest that the size of the micropores should be within a range of 2–5 µm when working with cells with sizes in the range of 10–25 µm. To perform cell trapping on the micropore array, a vacuum pressure between 7.5–12 psi would be optimal. A cell viability of more than 90% may be reached when using the vacuum pressure in this range^11^. In addition, chemical treatment may prevent cells from attaching to the chip surface so that cells can easily move along the chip surface to facilitate cell trapping by a low-pressure vacuum. The integration of a microfluidic system into the chip may also allow the recycling and repetitive loading of cells into chamber until all loaded cells are trapped by the micropores.

### Precise intracellular mRNA detection

**Fig. 2:**
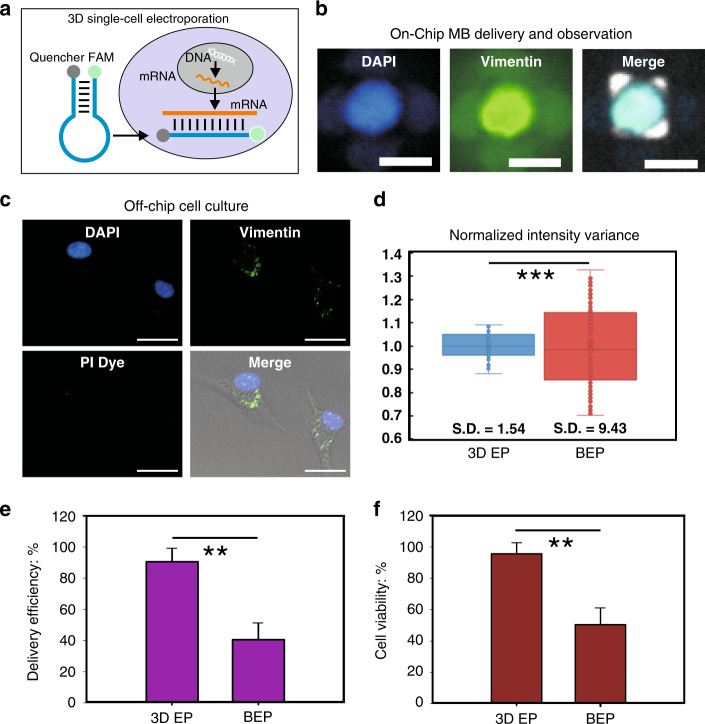
On-chip delivery of molecular beacons (MBs) for intracellular mRNA detection. **a** Vimentin CDS MBs were designed to probe the expression of mRNA to determine the regulation of the *VIM* gene in living cells. **b** The fluorescence observed at the single-cell level on the 3D EP chip indicates that vimentin is highly expressed in myofibroblasts. Scale bar: 5 µm. **c** Culturing myofibroblasts in Petri dishes verified the high expression of vimentin, as well as the presence of intact membranes after electroporation (PI dye). Scale bar: 20 µm. **d** The delivery uniformity of 3D EP chip and the commercial BEP, respectively. **e** The delivery efficiency of 3D EP chip and the commercial BEP, respectively. **f** The cell viability after electroporation with 3D EP chip or the commercial BEP, respectively. ***p-value < 0.005; **p-value < 0.01.

The mRNA detection in cells is a molecular analysis method that is commonly used in many research areas. The presence of myofibroblasts in heart tissue is an indication of cardiac fibrosis. However, there are limited numbers of markers used for identifying myofibroblasts^36^. To provide new insights into the intracellular markers used for characterizing myofibroblasts^37,38^, we designed molecular beacons (MBs)^39^ with the coding sequence (CDS) of the *VIM* (vimentin) gene, delivered them into myofibroblasts using micropore arrays, and analyzed the expression of vimentin in the cells (Fig. 2a).

On our platform, the FAM-labeled MBs were observed in cells trapped in micropores after electroporation, suggesting that the cells expressed the *VIM* gene (Fig. 2b). The cell membrane remained intact after recovery from electroporation, as shown by the impermeability of propidium iodide (PI) in the cells (Fig. 2c). Compared to a conventional electroporation system (BioRad Gene Pulser, BEP) that is commercially available, our 3D EP system provided better uniformity (*p* *<* 0.005, Fig. 2d). Furthermore, the 3D EP platform resulted in a higher delivery efficiency (90.5 vs. 40.6%, *p* *<* 0.01) and cell viability (95.6 vs. 50.4%, *p* *<* 0.01) (Fig. 2e, f).

### Dose-controllable delivery of chemo-drugs

**Fig. 3:**
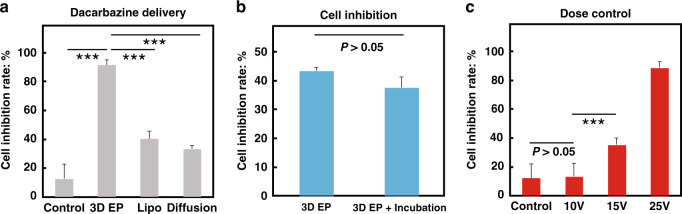
Dose-controllable chemo-drug delivery by using the 3D EP chip. **a** The inhibition rate in melanoma cells delivered with dacarbazine showed that the 3D EP system achieved a significantly higher cancer inhibition rate than the benchmark methods, including Lipofectamine and direct drug treatment (diffusion). ***p-value < 0.005. **b** No obvious difference was observed in the melanoma inhibition rates resulting from electroporation and poration followed by overnight incubation, which indicates that 3D EP-based drug delivery occurs via electroinjection. **c** Dose control of the chemo-drug can be achieved by changing the voltage of transfection. In the control group, the cells were incubated only with dacarbazine.

We also demonstrated that our 3D EP system may be used to inject small-molecule chemicals directly into cells. A chemotherapeutic agent, dacarbazine, which has been used to treat melanoma in the clinic, was tested. Human A375 melanoma cells treated with dacarbazine by using 3D EP system-mediated electroporation with a current pulse (25 V, 20 ms) showed a ~90% decrease in cell viability, in contrast to the control cells that received electroporation only (Fig. 3a). The exposure of A375 cells to either Lipofectamine-enveloped dacarbazine or dacarbazine alone led to a ~40% or less decrease in cell viability (Fig. 3a). We also tested the cell inhibition induced by 3D EP system-delivered dacarbazine using electroporation (15 V, 20 ms). No obvious difference in cell inhibition was observed between electroporated cells with and without overnight incubation in the drug-containing chamber (Fig. 3b), suggesting that drug delivery was achieved through electroinjection rather than electroporation-assisted diffusion across the cell membrane.

Another unique feature associated with 3D EP is the dose controllability. Compared to control cells without electroporation in the drug-containing chamber, a clear increase in cell death due to increased voltage was observed in cells subjected to electroporation (Fig. 3c). Meanwhile, the viability of the dacarbazine-free electroporated cells was above 90% and was similar to that of control cells that received no electroporation and dacarbazine treatment (Fig. S9). These results indicate that electroporation with our 3D EP system had negligible effect on cell viability. The reduction in the viability of cells subjected to 10 V electroporation was similar to that of the control cells (Fig. 3c).

### Macromolecular gene transfection

**Fig. 4:**
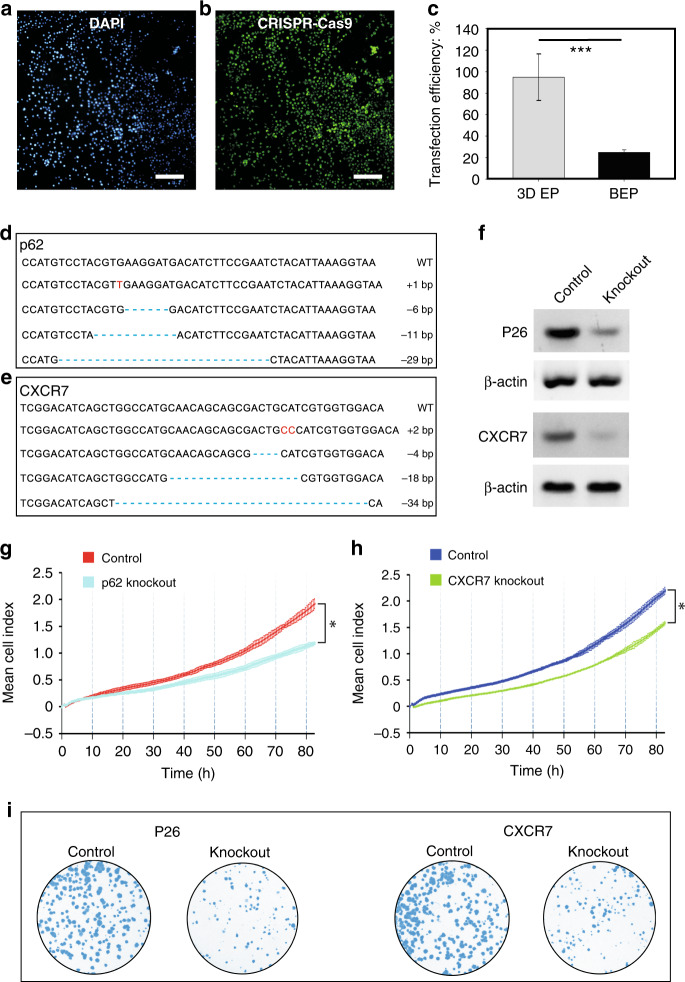
Application of our 3D EP system to CRISPR-Cas9 gene transfection and editing. **a** and **b** The 3D EP system achieved high-throughput gene transfection by delivering macromolecular CRISPR-Cas9 plasmids (>9 kb) into melanoma cells, offering a significantly higher efficiency than commercial BEP. Scale bar: 100 μm. **c** The transfection efficiency of CRISPR-Cas9 plasmids by 3D EP or BEP. **p-value < 0.01. **d** and **e** Sequencing results for the *p62* and *CXCR7* genes from CRISPR-Cas9 knockout cells transfected by using the 3D EP system. **f** Western blotting of *p62* protein and *CXCR7* protein from A375 cells in the knockout and control groups transfected by using the 3D EP chip. β-actin was used as a loading control. **g** and **h** Cell proliferation assay with the xCELLigence RTCA system. A total of 5 × 10^3^ cells were seeded in each well of an RTCA system plate followed by continuous real-time monitoring for 80 h. *p-value < 0.05. **i** Colony formation of A375 cells with or without gene knockout. Representative dishes from at least three replicate experiments stained by Giemsa stain were shown.

The CRISPR-Cas9 system represents a promising approach for gene editing^40,41^. Similar to other methods used for the expression of exogenous genes, CRISPR-Cas9-mediated gene editing is frequently enabled through the delivery of DNA vectors that express the Cas9 protein and sgRNA into cells. Normally, a DNA plasmid consists of thousands of nucleotides that cause the entire macromolecule to be highly negatively charged^42^. To test the potential use of our 3D EP system for the transfection of plasmid DNA for CRISPR-Cas9-mediated gene editing, we used commercially available DNA plasmids that carry a GFP (green fluorescent protein) reporter gene. Melanoma is a skin cancer that may be treated by utilizing a platform for gene editing in vivo. Using melanoma cells as a model, we delivered CRISPR-Cas9 plasmids as cargo into A375 cells with the 3D EP platform (Fig. S10). The expression of GFP confirmed that our platform achieved the successful delivery of the macromolecular plasmids (>9 kb) (Fig. 4a, b**)**. Compared with conventional BEP (~20%), our platform can achieve much higher efficiency (>90%) (Fig. 4c).

Furthermore, we demonstrated the performance of the system for gene editing and tumor inhibition by transfecting CRISPR-Cas9 plasmids. Two CRISPR-Cas9 plasmids were directly delivered into A375 cells to knock out the *p62* and chemokine receptor *CXCR7* genes. These two genes have been proven to promote cell proliferation during tumor development^43,44^. The PCR (polymerase chain reaction) products from the genomic DNA of the transfected cells were sequenced. Representative sequencing results indicated that the sites of the *p62* and *CXCR7* genes were successfully edited (Fig. 4d, e). The protein abundances of *p62* and *CXCR7* were examined by Western blot (Fig. 4f**)**. As expected, the knockout cells clearly expressed lower levels of target proteins compared to those in control cells. To test whether the loss of genes would affect cancer cell growth, we monitored the cells with an electrical impedance-based xCELLigence RTCA system that allows the long-term real-time recording of live cell proliferation in a noninvasive manner^45,46^. The deficiency of either *p62* or *CXCR7* significantly decreased the proliferation of cells during continuous monitoring for 80 h (Fig. 4g, h). A colony formation assay further confirmed the reduced growth of the cells without *p62* or *CXCR7* (Fig. 4i). These results demonstrated that our system enables effective gene editing and tumor therapy by direct CRISPR-Cas9 delivery.

## Conclusion

Electroporation has been widely used for gene transfection and drug delivery. However, recent years have witnessed the limits of their application both in vitro and in vivo, especially for cargoes with large molecular weights. On the other hand, conventional electroporation systems show limited control of transfection efficiency, cell viability and uniformity at the single-cell level. In this work, we designed a 3D micropore array platform for multiplexed single cell patterning and low-voltage electroporation. We fabricated a silicon-based pyramid pit micropore array chip using a simple wet-etching protocol. The pyramid pit shape provided an isolated cell culture environment where in situ single-cell transfection and gene expression investigation can be achieved. A simple yet rapid vacuum setup was established to trap cells on the micropore array. We conducted trapping optimization by considering some critical factors, including the micropore diameter, negative pressure level, and chip surface properties. A variety of cargoes were selected to demonstrate the delivery/transfection outcomes for the 3D electroporation platform. The device showed significantly higher uniformity, delivery efficiency and cell viability than the commercial electroporation system. In addition, we observed that the device offered direct-injection and dose-controllable method for the delivery of chemo-drugs (dacarbazine) into cancer cells (melanoma), which produced a significantly higher cancer inhibition rate than Lipofectamine. Finally, the device showed high efficiency in transfecting CRISPR-Cas9 plasmids (>9 kb) and effective tumor inhibition by CRISPR-Cas9-induced gene editing. Overall, 3D electroporation is a high-throughput, uniform and safe nonviral delivery method in vitro. The system extends the application of electroporation to living cell gene expression investigation, cancer therapy with chemo-drugs and gene editing.

## Methods and materials

### Cell preparation

Three types of cell lines were used in this work. In the cell trapping experiments, we used BEAS-2B cells (human bronchial epithelium, ATCC) because of their round morphology and good uniformity in terms cell size (~25 μm), and they presented an ideal cell model for investigating cell trapping efficiency. In the experiments involving intracellular investigation in vitro, there was a critical need to identify myofibroblasts markers to provide new insights into the development of cardiac fibrosis. Therefore, we chose human cardiac fibroblasts (HCF-a, Catalog No. 6320, ScienCell Research Laboratories) for molecular beacon delivery and gene detection. Human melanoma (A375) cells (CRL-1619) purchased from ATCC were used for chemo-drug delivery and CRISPR-Cas9 transfection, which could be further applied to cargo delivery in vivo.

BEAS-2B cells were cultured in 25 cm^2^ T-flasks (Corning, Sigma-Aldrich) incubated at 37 °C in an atmosphere with 5% CO_2_, with nutrient medium consisting of RPMI 1640 (Gibco, catalog No. 11875–093) containing 10% (v/v) newborn calf serum (NCS, heat-inactivated, catalog No. 26010). Cardiac fibroblasts and A375 cells were cultured in 25 cm^2^ flasks or 6-well cell culture plates (Costar, NY, US) with DMEM (Gibco) supplemented with 15% ES-Cult fetal bovine serum (FBS, StemCell Technologies, catalog No. 06952).

The cell culture medium was replaced with fresh medium every 3–5 days, and cell passaging was carried out every 5~7 days once the cells reached 80% confluency. Cells were detached from the T-flasks by incubating them for 3 min with 1 ml 0.25% trypsin-EDTA (1×) (Life Technologies, NY, US, catalog No. 25200). The cells were precipitated by centrifugation at 1200 rpm for 4 min and resuspended in fresh medium at 1 × 10^5^ cells/ml.

### Bioreagents

Hoechst stain (Sigma-Aldrich, catalog No. 654434, excitation/emitting wavelength, 350/461 nm) was used to specifically stain the cell nucleus. Before trapping, 2 μl Hoechst stain (50 μg/ml) was added to 1 ml of cell suspension (1 × 10^5^ cells/ml), followed by 10 min of incubation. DAPI (Sigma-Aldrich, CAS No. 28718–90–3) was used with the same purpose as Hoechst stain. Calcein acetomethoxy fluorescein complex (Calcein AM, catalog No. C3099, 1 mg/ml, Invitrogen, excitation/emitting wavelength, 495/515 nm) was used to test cell viability after cell trapping. Propidium iodide (PI, catalog No. P3566, Invitrogen) was prepared to evaluate cell membrane damage induced by vacuum or electroporation. The fluorescence was excited at 535 nm, while the maximum emission was at 617 nm (red fluorescence).

Molecular beacons (MBs) were synthesized by Biosearch Technologies (CA, USA). We used the OligoArchitectTM Online tool (http://www.oligoarchitect.com/ShowToolServlet?TYPE=BEACONPROBE) for the molecular beacon design. The specific mRNA sequence was determined by using PubMed (https://www.ncbi.nlm.nih.gov/gene/). The designed MB nucleotide sequence is given below:

vimentin MB: CGCGATCCGCGAUGCAGGCGGCCAAUAGUGUGAUCGCGGATCGCG

The chemo-drug dacarbazine was provided by Dr. Yu-Chieh Wang at the University of North Texas Medical Center. For electroporation, the drugs were prepared in PBS solution (pH 7.4) at a concentration of 10 µM. The CRISPR-Cas9 plasmids (9 kb, SCBT, Cat. No. SC-400504) encoding the GFP reporter were purchased from Addgene. For the benchmark control method, Lipofectamine 2000 (Cat. No. 11668207, ThermoFisher) was used to deliver dacarbazine into A375 cells.

### Micropore array chip fabrication

The silicon chip was fabricated based on silicon anisotropic wet-etching with six major steps (see Fig. S5). A silicon wafer (crystal plane (100), DSP, 300 μm thick, purchased from University Wafer) was coated with a 100 nm-thick SiO_2_ film by PECVD (Plasma Therm 790), followed by standard photolithography (EVG 620, EVG group Inc, NY) and reactive ion etching (RIE, CF_4_). An etching film mask (SiO_2_) with a square array pattern (side length: 1 mm) was used on one side of the silicon wafer (denoted back side). The silicon area without protection, including the top side, was etched in a 45% potassium hydroxide (KOH) bench tank at 80 °C. The stop time was accurately controlled, ensuring that the thickness in the central area (square region) was thinned to ~35 μm. Then, the back side was coated with another 100 nm-thick SiO_2_ film by PECVD for etching protection. A thin metal layer (Cr/Au, 30 nm/100 nm) was coated on the top side of the silicon chip by E-beam evaporation. The pattern of the microsquare array (side length: 50 μm, center-to-center distance: 75 μm) was transferred to the Cr/Au metal layer by selective etching with chromium etchant (CR-7S, Cyantek Com., CA, US) and gold etchant (GE-8111, Transene Company Inc., MA, US). Using Cr/Au as the top side mask, we etched the top side using KOH wet etching to forming the pyramid pit array. The size of the opening at the bottom of the pyramid pit was controlled by the stop time. Finally, the Cr/Au layer was removed. A scanning electron microscope (Hitachi S-3000) was used to characterize the morphologies of the fabricated microchannel arrays, as well as the sizes of the open pores.

### Vacuum-assisted cell patterning

We used a vacuum method approach to load cells onto the microchannel array by using the experimental setup shown in Fig. S6. A porous cork with high porosity was mounted on a filter bottle, which had a nozzle linked to a vacuum pump. The vacuum pressure was controlled by a valve. Once the chip was placed on the porous cork, the vacuum constantly aspirated air into the filter bottle through the micropore array of the silicon chip, generating negative pressure. When a droplet of cell buffer with a certain cell number was placed on the chip, the negative pressure sucked the solution into the filter bottle through every micropore, and the cells were hydrodynamically driven towards the micropore array and were locked into each micropore with tight trapping. To avoid cell loss, a chamber was made on the boundaries of the silicon chip after mounting a polydimethylsiloxane (PDMS) spacer. The fabricated silicon chip was cut into a 1 cm square.

In the next step of the chip surface treatment, each silicon chip was placed in a 12-well plate. One milliliter of gelatin solution was added to each well, completely covering the chip. The plate was placed in an incubator (37 °C, 5% CO_2_) for 1 h and stored at 4 °C in a cold room before usage. A 1 ml BSA (4%) solution was prepared in Tris buffer and added to the well. The albumin was passively absorbed onto the silicon surface. The total time of absorption was half an hour before the vacuum experiment. For PEG treatment, the silicon chip was first coated with 100 nm of gold with electron beam evaporation before PEG treatment. The silicon chip was immersed in PEG solution for 1 h in an incubator (37 °C, 5% CO_2_) before the vacuum experiment.

### 3D EP setup

The 3D electroporation system consisted of a supportive platform (polymethylmethacrylate, fabricated by a high-precision digital machine, AeroTech Inc., Pittsburgh, PA), a bottom electrode (Cr/Au, 30 nm/100 nm, deposited on a microscope slide with electron beam evaporation), a top electrode (gold-coated glass slide), and other components (Fig. 1c). The supportive platform was the core element and had the major function of holding the chip with trapped cells during electroporation. The platform was compartmentalized into two chambers: a lower chamber (6 mm × 6 mm × 1 mm) and an upper chamber (10 mm × 10 mm × 2 mm). A PDMS spacer with the same dimensions as the holding platform and bottom electrode deposited onto the slide was designed to bond the two components together to avoid liquid leakage when the transfection reagent solution was injected into the lower chamber. All components were assembled on a PMMA foundation with two clamps.

Before electroporation, the system was assembled and sterilized with 70% ethanol, followed by UV exposure for 10 min. A piece of clear tape was applied to the edges of the bottom and the top chamber to tightly seal the cell-entrapping chip, enabling the electric field to pass through the micropores during cell poration. A total of 1 ml of cell buffer was placed in the top chamber for culturing the cells, followed by the injection of the cargo into the lower chamber through the inlet. Electroporation was conducted between the top electrode, which was placed in the top chamber, and the bottom electrode by using a commercial power supply (BioRad Gene Pulser). The entire process of cell trapping and electroporation required only 5 min.

### CRISPR-Cas9 plasmid transfection and verification

After the A375 cells were transfected with the CRISPR-Cas9 plasmid by the vacuum-assisted 3D EP platform, the cells were further cultured in an incubator for 48 h. Subsequently, the expression of the fluorescent protein was observed under a confocal microscope (FV1000 Spectral Confocal, Olympus). The *p62* and *CXCR7* genes in the genomic DNA of the transfected A375 cells were amplified by PCR and sequenced. To verify the expression of the proteins, A375 cells were lysed and subjected to Western blotting. In addition, the cell proliferation ability was examined using a Real-Time Cell Analysis (RTCA) system (RTCA DP Instrument; ACEA Biosciences, Inc., USA). For the long-term monitoring of cellular proliferation, cells were seeded onto RTCA E-plates at a density of 5 × 10^3^ cells per well. The electrical impedance in each well was continuously measured for 80 h. The change in electrical impedance caused by the cell cycle and cell division was recorded and converted into an index of cell proliferation by the RTCA program. For the colony formation assay, A375 cells were seeded in 6-well plates at a density of 500 cells per well. After two weeks of culture at 37 °C in 5% CO_2_, the cells were fixed in 4% paraformaldehyde for 30 min and stained with Giemsa staining solution for 20 min followed by washing. The colony formation assay was repeated at least three times.

### Image acquisition and data analysis

A fluorescence microscope (Nikon Eclipse Ti, Nikon Instruments Inc.) was used to check the cell trapping and delivery results. Nikon Ti Element software was used for statistical analysis. A confocal microscope was used to study the trapping of cell bodies inside the micropores by the vacuum. A 3D cell model was drawn based on fluorescence from FAM and Hoechst stain (FV10-ASW 2.0 Viewer, Olympus).

The living cells were stained with AM (green fluorescent dye), while the dead cells were stained with PI (red fluorescent dye). The transfected cells were successfully transfected with a plasmid containing green fluorescent protein (GFP). The total number of cells could be determined by nuclear staining (DAPI, blue fluorescence). Therefore, the number of cells could be determined by imaging and counting using fluorescence microscopy.

The cell viability, inhibition efficiency and transfection efficiency were calculated based on the following equations:

$${\mathrm{Cell}}\,{\mathrm{viability}} = \frac{{{\mathrm{The}}\,{\mathrm{number}}\,{\mathrm{of}}\,{\mathrm{living}}\,{\mathrm{cells}}}}{{{\mathrm{The}}\,{\mathrm{number}}\,{\mathrm{of}}\,{\mathrm{total}}\,{\mathrm{cells}}}}\,\%$$

$${\mathrm{Cell}}\,{\mathrm{inhibition}}\,{\mathrm{efficiency}} = \frac{{{\mathrm{The}}\,{\mathrm{number}}\,{\mathrm{of}}\,{\mathrm{dead}}\,{\mathrm{cells}}}}{{{\mathrm{The}}\,{\mathrm{number}}\,{\mathrm{of}}\,{\mathrm{total}}\,{\mathrm{cells}}}}\,\%$$

$${\mathrm{Gene}}\,{\mathrm{transfection}}\,{\mathrm{efficiency}} = \frac{{{\mathrm{The}}\,{\mathrm{number}}\,{\mathrm{of}}\,{\mathrm{transfected}}\,{\mathrm{cells}}}}{{{\mathrm{The}}\,{\mathrm{number}}\,{\mathrm{of}}\,{\mathrm{total}}\,{\mathrm{cells}}}}\,\%$$

The force experienced by a single cell was calculated according to the basic force model: F = P × S (F: force, P: pressure, S: force area). Due to the flexibility of the cells, the force area of the cells may account for half of the cell surface area and could be determined with the equation force area S = 4πR^2^/2 (R: the radius of cell). The pressure exerted on each cell could be regarded as measurable and uniform. The force applied to a single cell is represented by F = 2πR^2^ × P. When the diameter of the cell was 20 μm and the applied pressure was 7.5 psi (1 psi = 6.90 kPa), the force on a single cell could be calculated. Therefore, the force experienced by a single cell was 3.25 × 10^−5^ N when the applied pressure was 7.5 psi.

All experiments were performed in triplicate. Statistical analyses were performed in SigmaPlot version 13.0. Comparisons between groups were made by analysis of variance (ANOVA). Differences with *p*-values < 0.05 were considered statistically significant.

## Supplementary information

Supporting Information File

Graphical abstract
