# Supplementary material for: On-chip multiplexed single-cell patterning and controllable intracellular delivery
Source: Microsyst Nanoeng. 2020 Feb 24;6:2. doi: 10.1038/s41378-019-0112-z (PMC8433345; doi:10.1038/s41378-019-0112-z)
Supplement: Supplementary file 1 — Supporting Information File [file 41378_2019_112_MOESM1_ESM.docx]

**On-Chip Multiplexed Single-Cell Pattern and Controllable Intracellular Delivery**

Zaizai Dong^1,2#^, Yanli Jiao^3#^, Bingteng Xie^4^, Yongcun Hao^5^, Pan Wang^4^, Yuanyuan Liu^1,2^, Junfeng Shi^6^, Weiwei An^1,2^, Chandani Chitrakar^7^, Stephen Black^7^, Yu-Chieh Wang^8^, L. James Lee^6^, Mo Li^4*^, Yubo Fan^1,2^, Lingqian Chang^1,2,7*^

1 School of Biological Science and Medical Engineering, Beihang University, Beijing, 100083, China

2 Institute of Nanotechnology for Single Cell Analysis (INSCA), Beijing Advanced Innovation Center for Biomedical Engineering, Beihang University, Beijing, 100083, China

3 College of Agricultural and Life Science, University of Florida, Gainesville, FL, 32611, USA

4 Center for Reproductive Medicine, Peking University Third Hospital, 100191, Beijing, China

5 Ministry of Education Key Laboratory of Micro and Nano Systems for Aerospace, Northwestern Polytechnical University, Xi’an, 710072, China

6 Chemical and Biomolecular Engineering Department, Ohio State University, Columbus, OH 43209, USA

7 Department of Biomedical Engineering, University of North Texas, Denton, TX, 76207, USA

8 Department of Pharmaceutical Sciences, University of North Texas Health Science Center, Fort Worth, TX, 76107, USA

* Corresponding authors: [changlingqian1986@buaa.edu.cn](mailto:changlingqian1986@buaa.edu.cn); [limo@hsc.pku.edu.cn](mailto:limo@hsc.pku.edu.cn)

# These authors contribute equally.


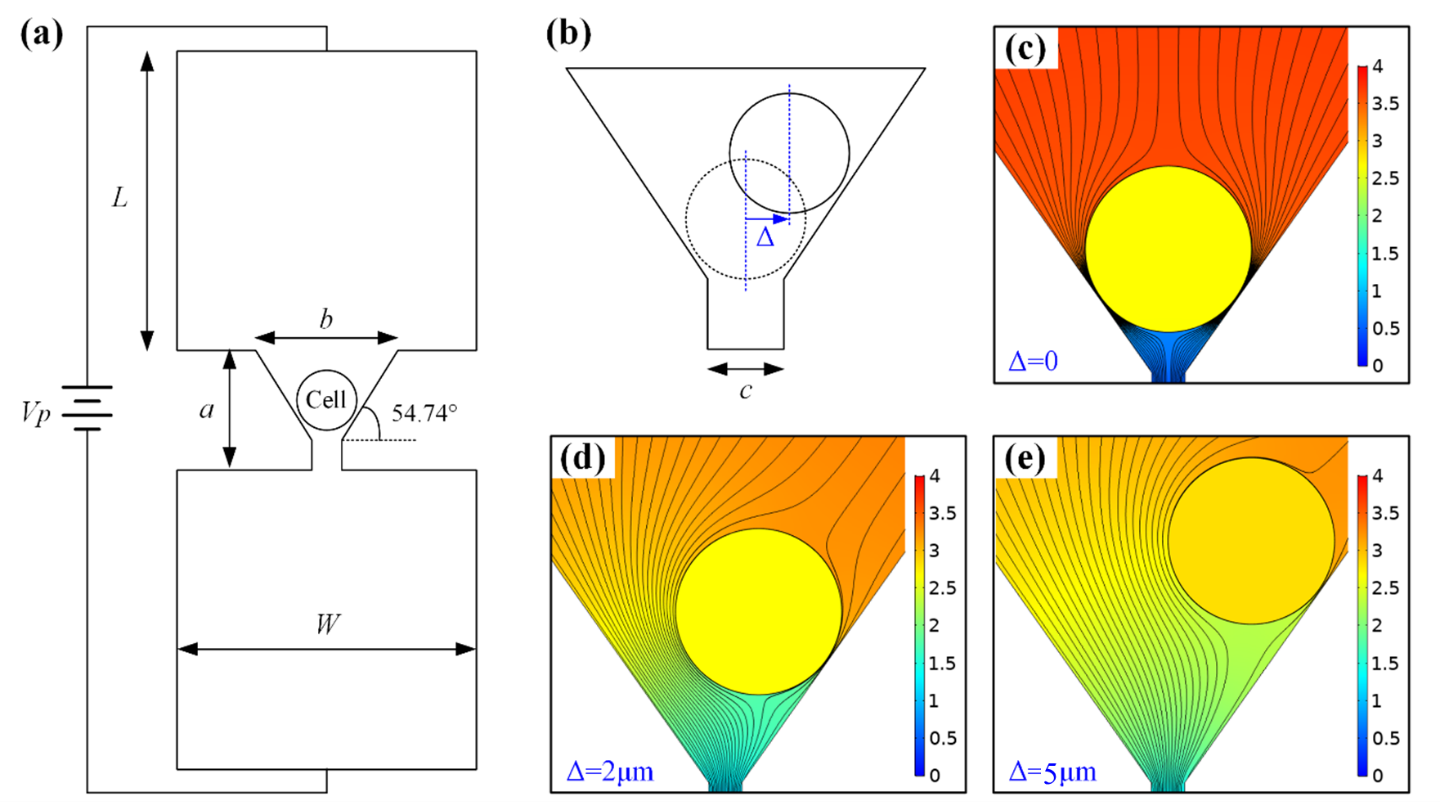


**Figure S1.** **Simulation of cell electroporation for pyramid pit**. (a) Simulation parameters for one set of cell electroporation. L: reservoir depth (100μm), W: cell-to-cell distance (100μm), a: reservoir space (35μm), b: pyramid pit width (50μm) and Vp: applied voltage (4V). The cell diameter is 10μm. (b) The zoomed in pyramid pit area. c: micropore diameter (2μm) and Δ: distance between cell center and micropore central line. (c)-(e) Simulated electrical potential contours and electrical field streamlines around the cell for Δ=0, 2μm and 5μm, respectively.


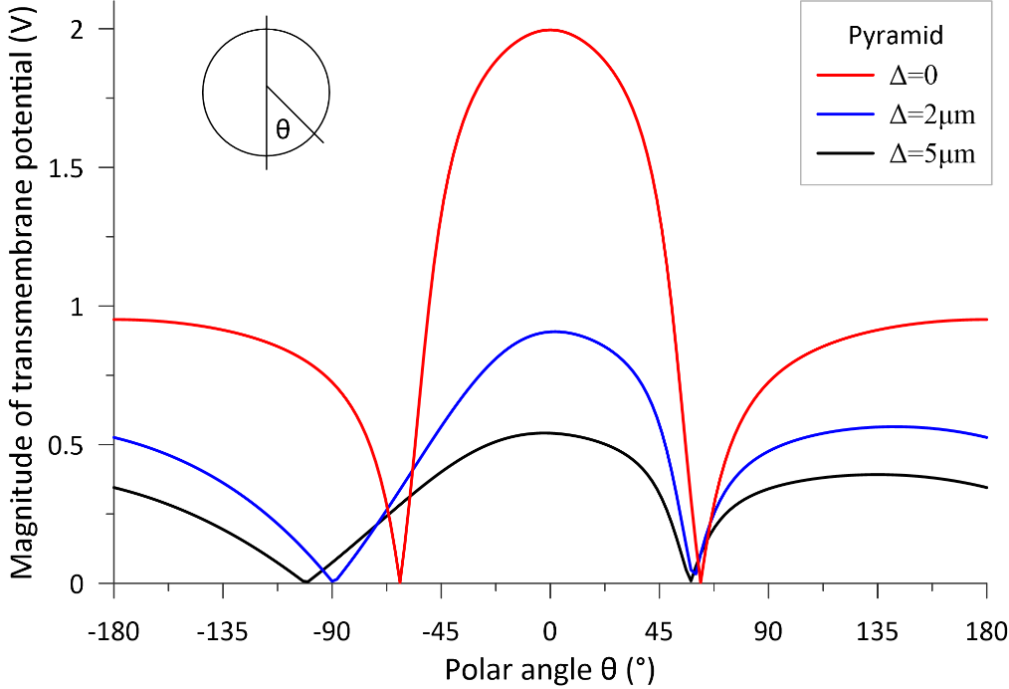


**Figure S2.** **Simulated transmembrane potential magnitudes for pyramid pit.** The simulation of transmembrane potential when Δ=0, 2μm and 5μm, respectively. Δ: distance between cell center and micropore central line. Polar angle θ is defined with respect to the Y-axis.


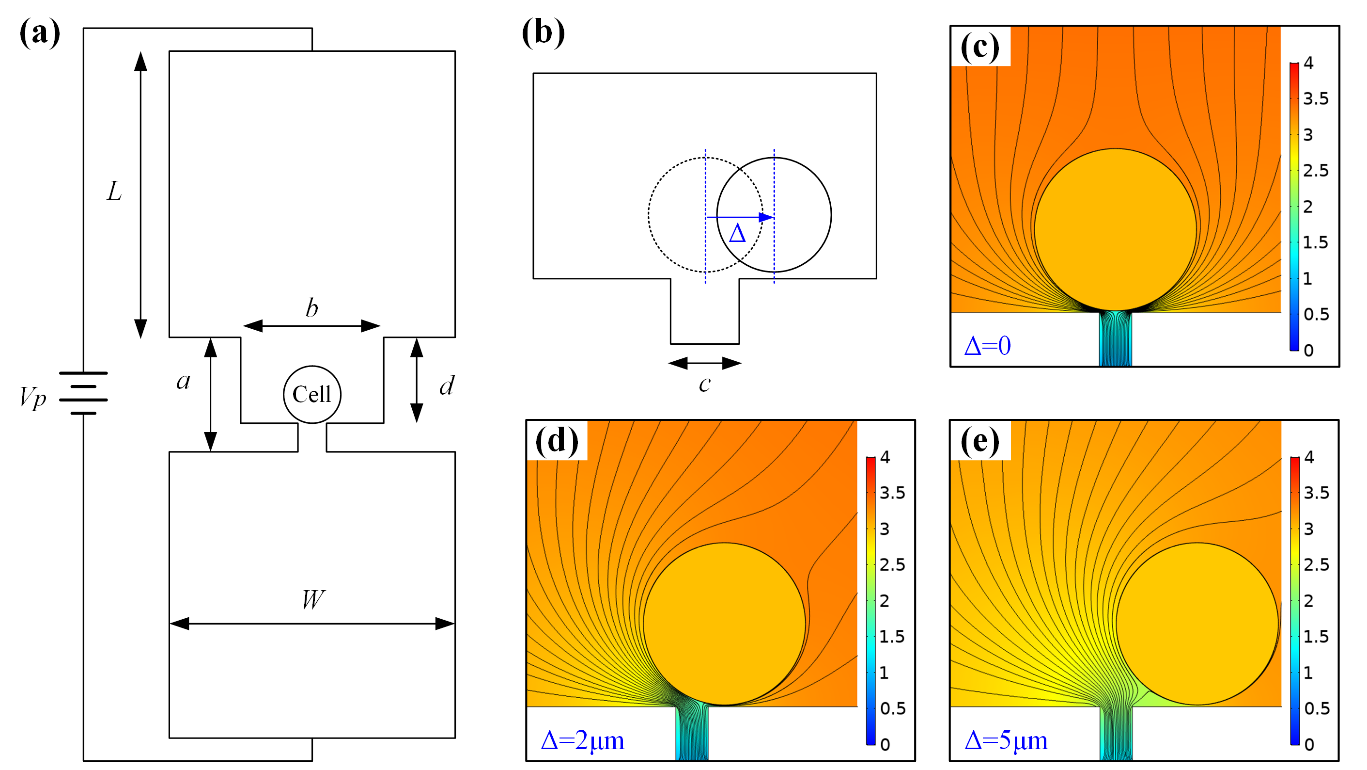


**Figure S3.** **Simulation of cell electroporation for** **flat support.** (a) Simulation parameters for one set of cell electroporation. L: reservoir depth (100μm), W: cell-to-cell distance (100μm), a: reservoir space (35μm), b: flat support width (50μm), d: flat support depth (32μm) and Vp: applied voltage (4V). The cell diameter is 10μm. (b) The zoomed in flat support area. c: micropore diameter (2μm) and Δ: distance between cell center and micropore central line. (c)-(e) Simulated electrical potential contours and electrical field streamlines around the cell for Δ=0, 2μm and 5μm, respectively.

**
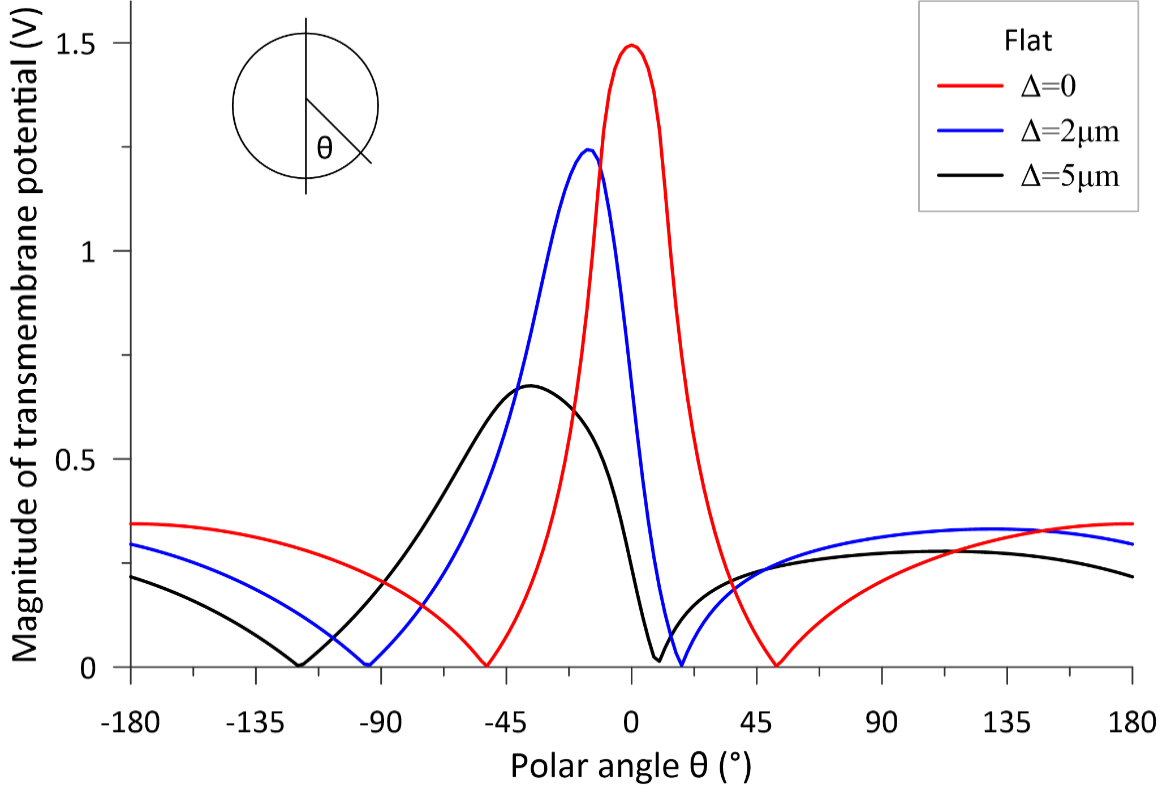
**

**Figure S4. Simulated transmembrane potential magnitudes for flat support.** The simulation of transmembrane potential when Δ=0, 2μm and 5μm, respectively. Δ: distance between cell center and micropore central line. Polar angle θ is defined with respect to the Y-axis.

**Figure S5. The fabrication protocol of the pyramid pit shaped micropore array chip, mainly based on silicon anisotropic wet etching.**

**Figure S6. The experimental setup and working mechanism of the vacuum-assisted cell trapping on micropore array chip.** The valve controlled the negative pressure generated within the chamber. The value of negative pressure was read out by a gauge installed on the valve.

**Figure S7. Comparing cell trapping efficiencies on the micropore array chip modified with three chemicals, i.e. gelatin, BSA and PEG, respectively.** Vacuum (negative pressure) was adjusted while the ratio of loaded cell number to micropore number was fixed at 1: 1. The pore size is 2 μm.

**Figure S8. Investigation on the vacuum induced cell damage in correspondence to different micropore size.** (a) The percentage of cell body inside the micropore with different pore size. (b) The cell viability of trapped cells on the micropores with different pore size.

**Figure S9. Effect of** **electroporation on cell viability.** (a) The rate of cell viability is >95% after electroporation by different voltages. Control group was not performed electroporation. (b) Confocal image of cells after electroporation under 25V (blue-DAPI-cell nucleus, red-PI-dead cells). Cell viability = (1-the number of dead cells/ the number of total cells) %

**Figure S10. Delivery of CRISPR-Cas9 DNA plasmids.** Delivered into melanoma cells by 3D EP platform, the mechanism of CRISPR-Cas9 on gene knockout and downregulate the *p62* and *CXCR7* protein.
