# Supplementary figures and images for: On-chip multiplexed single-cell patterning and controllable intracellular delivery
Source: Microsyst Nanoeng. 2020 Feb 24;6:2. doi: 10.1038/s41378-019-0112-z (PMC8433345; doi:10.1038/s41378-019-0112-z)

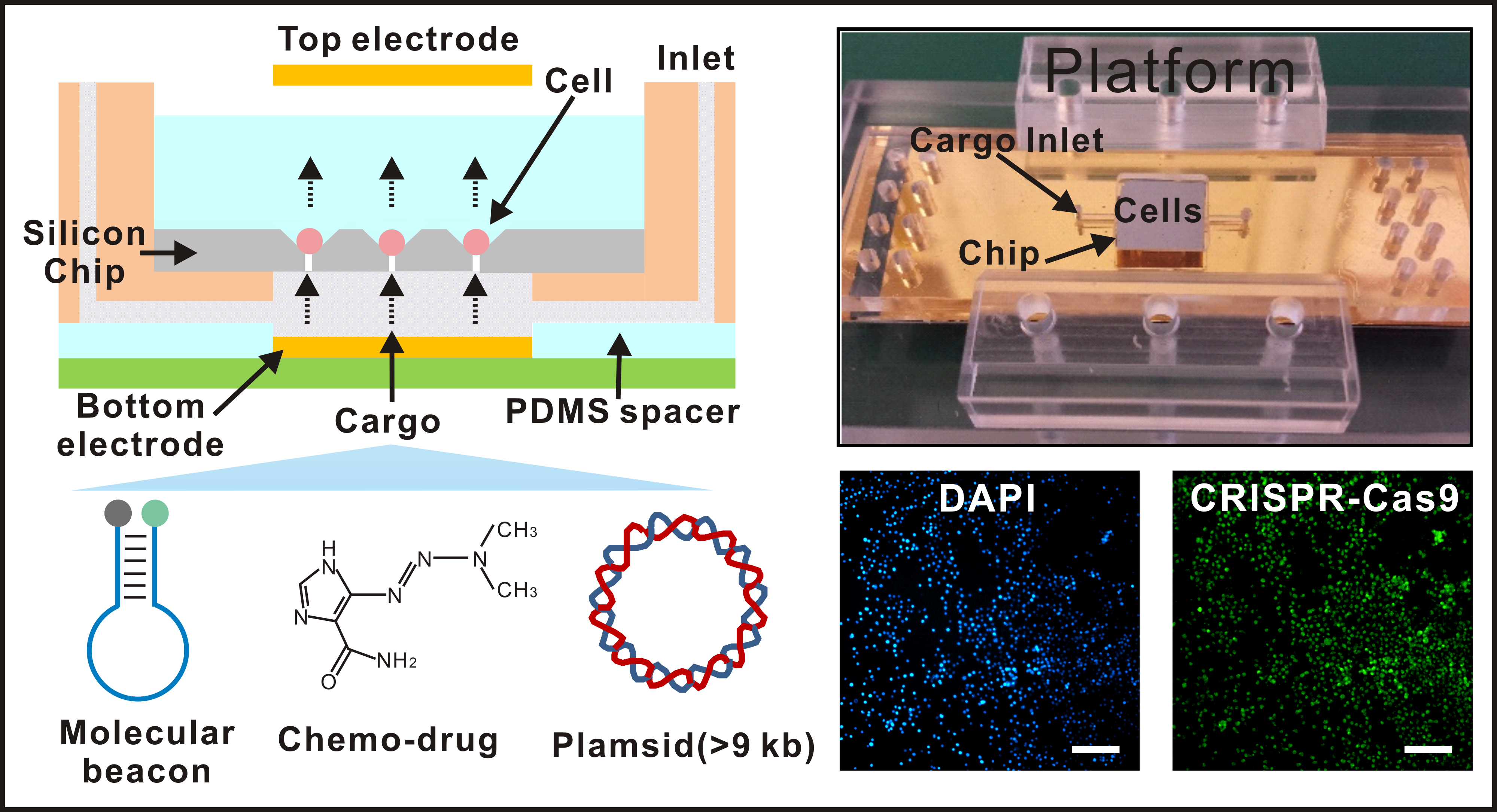

Supplement: Supplementary file 2 — Graphical abstract [file 41378_2019_112_MOESM2_ESM.tif]
